# A human skeletal muscle cross‐bridge model to characterize the role of metabolite accumulation in muscle fatigue

**DOI:** 10.1113/EP092843

**Published:** 2025-05-31

**Authors:** John I. Hendry, Muhammet Enes Erol, Gwenael Layec, Edward P. Debold, Shivendra G. Tewari, Anders Wallqvist, Venkat R. Pannala

**Affiliations:** ^1^ Department of Defense Biotechnology High Performance Computing Software Applications Institute Defense Health Agency Research & Development, Medical Research and Development Command Fort Detrick Maryland USA; ^2^ The Henry M. Jackson Foundation for the Advancement of Military Medicine, Inc. Bethesda Maryland USA; ^3^ Department of Kinesiology University of Massachusetts Amherst Massachusetts USA; ^4^ School of Health and Kinesiology University of Nebraska Omaha Nebraska USA

**Keywords:** cross‐bridge cycle, muscle force generation, musculoskeletal modelling, plantar flexion, skeletal muscle fatigue

## Abstract

Skeletal muscle fatigue is accompanied by the accumulation of metabolites, such as adenosine diphosphate (ADP), inorganic phosphate (P_i_), and protons (H^+^). However, we lack a comprehensive understanding of the contribution of these metabolic changes to the development of muscle fatigue during intense exercise and the underlying mechanisms. To address this gap, we collected data from young adults performing a dynamic (0.75 Hz) plantar flexion exercise to task failure (642 ± 104 s), including in vivo concentrations of metabolites and H^+^ measured by ^31^P magnetic resonance spectroscopy as well as muscle activation signals obtained via electromyography. Using these data, we developed and validated a human skeletal muscle model. Our model‐based simulations suggested that to continue the plantar flexion exercise at the required power output, muscle activation should progressively increase. In the absence of this increased activation, we observed a reduction in force‐generating capacity due to metabolite‐mediated inhibition of actin–myosin cross‐bridge cycling. Our simulations also showed that P_i_ reduced force production by 30% when we increased it 50% above the concentrations measured experimentally. A parameter sensitivity analysis suggested that force generation is strongly dependent on the rate of P_i_ release from the actin–myosin complex, and P_i_ inhibits force by increasing the rate of actin–myosin detachment. In addition, we proposed an alternative mechanism through which H^+^ might reduce muscle force generation during exercise. In contrast, elevated ADP levels did not significantly affect force generation. This study provides insight into the impact of metabolite accumulation on force generation and muscle fatigue development.

## Abstract (highlights)

- **What is the central question of this study?**
  Force generation in skeletal muscles is driven by ATP hydrolysis and thereby results in the accumulation of its metabolite byproducts, such as ADP, P_i_ and H^+^, which can lead to muscle fatigue during intense exercise: what are the individual contributions of each of these metabolites to muscle fatigue development and the underlying mechanisms?
- **What is the main finding and its importance?**
  Using ^31^P‐MRS and electromyography data from exercising humans and a computational model, we demonstrate that P_i_ accumulation inhibits force generation by hindering actin–myosin cross‐bridge cycling during intense exercise. The developed skeletal muscle model helps in understanding the role of metabolite accumulation in muscle fatigue development.

## 1 INTRODUCTION

Muscle fatigue, in the context of intense contractile activity, is defined as the inability to sustain the force or power output required to complete a task following a prolonged muscular activity (Alba‐Jimenez et al., 2022; Constantin‐Teodosiu & Constantin, 2021; Edwards, 1981; Fitts, 1994). Physiologically, muscle fatigue can be the combined outcome of a complex interplay of several cellular events, including changes in neuromuscular metabolism, structural modifications, lowered oxygen/nutrient supply, and a compromised efficiency of the neuromuscular system to recruit additional motor units (Kay et al., 2001; Pethick et al., 2021). In particular, changes in skeletal muscle metabolism can directly impact the actin–myosin cross‐bridge cycle that lies at the heart of muscle contraction, thereby resulting in the inability of muscles to generate the required force to continue an activity (Allen et al., 2008; Debold, 2012; Sundberg & Fitts, 2019). In skeletal muscle cells, such metabolic changes may be the direct and immediate outcome of muscle contraction due to the energy demands associated with the process (Barclay, 2017). Actin–myosin sliding is powered by adenosine triphosphate (ATP) hydrolysis, resulting in the accumulation of adenosine diphosphate (ADP), inorganic phosphate (P_i_), and protons (H^+^) during intense contractile activity. This process is simultaneously accompanied by the accumulation and depletion of creatine (Cr) and phosphocreatine (PCr), respectively, due to the ATP buffering activity of the creatine kinase enzyme. Depending on the intensity of the contractile activity and the available oxygen supply, glycolysis and oxidative phosphorylation may also be activated to replenish ATP, resulting in further changes in intramuscular metabolite levels. Although the individual effects of these metabolite alterations during exercise are well documented, there still exists considerable ambiguity as to how they impact the force‐generation capacity.

Non‐invasive techniques, such as ^31^P magnetic resonance spectroscopy (^31^P‐MRS), have allowed us to quantify intramuscular metabolic changes during an intense physical activity performed by humans (Kemp et al., 2007; Meyerspeer et al., 2020; Shenton et al., 1986). In general, during an intense exercise, P_i_ levels increase from 3 to 5 mM in the resting state to >30 mM (Broxterman et al., 2017; Kemp et al., 2007). Similarly, ADP levels increase from ∼5 to 10 µM in the resting state to 0.2 mM under fatigue (Cooke, 2007). Similarly, due to an increase in H^+^ concentration, pH has been reported to drop as low as 6.2 (Broxterman et al., 2017; Cady et al., 1989; Wilson et al., 1988). Interestingly, ATP levels remain reasonably stable, dropping only by ∼20% in severely depleted muscles (Greenhaff et al., 1994), owing to the ATP buffering activity of creatine kinase and the activation of ATP‐generating pathways, such as glycolysis and oxidative phosphorylation (Hargreaves & Spriet, 2020; Sundberg & Fitts, 2019). Understanding the contribution of these metabolite alterations to muscle fatigue development and its underlying mechanisms has garnered significant scientific interest as evidenced by numerous studies over the last century. A recent detailed review of these works can be found in Sundberg and Fitts (2019). Several studies with rat, rabbit and human muscles have shown that accumulation of P_i_ and H^+^ negatively impacts force generation in skeletal muscle fibres both individually (Coupland et al., 2001; Debold et al., 2004; Knuth et al., 2006; Pate et al., 1995; Sundberg et al., 2018) and synergistically (Karatzaferi et al., 2008; Nelson et al., 2014). However, these studies were conducted with isolated muscle fibres under in vitro conditions and may not represent the human in vivo microenvironment under intense exercise. Therefore, we need a systematic study of the effects of these factors using data that capture, in real time, the alterations in intramuscular metabolite levels in exercising humans. The practicality of obtaining such data has been demonstrated in earlier studies (Broxterman et al., 2017; Sundberg et al., 2019).

Use of computational models can shed light on the mechanisms through which these metabolites impact force production and can quantify their potential contribution to force inhibition and muscle fatigue development (Debold et al., 2011, 2013). Currently, there are several computational models in the literature that describe muscle force generation, but they do not completely account for the interplay between several components involved in the process. For example, there are computational models that describe the actin–myosin cross‐bridge cycle (Herzog & Schappacher‐Tilp, 2023; Walcott et al., 2012), skeletal muscle metabolism (Lai et al., 2008; Lambeth & Kushmerick, 2002; Lopez et al., 2020), and metabolite‐mediated modulation of cross‐bridge kinetics (Pate & Cooke, 1989; Tewari et al., 2016). However, combining these models would provide a unique opportunity to develop a comprehensive model that accounts for all aspects of skeletal muscle force generation. Furthermore, these models would be most effective if calibrated using experimental data on alterations in intramuscular metabolite levels, power and muscle activation collected from humans. Indeed, advances in non‐invasive techniques, such as ^31^P‐MRS (Kemp et al., 2007; Meyerspeer et al., 2020), surface electromyography (EMG) (Li et al., 2024; Sun et al., 2022) and ergometers, have enabled the generation of such datasets for different exercise protocols (Broxterman et al., 2017; Hureau et al., 2022; Layec et al., 2009), yet to the best of our knowledge, currently there are no such concerted studies reported in the literature that combine all these methods together. Such an interdisciplinary (computational/experimental) approach would not only provide a system‐level understanding of muscle fatigue development but also act as a test bed to evaluate existing hypotheses on muscle fatigue development.

In this study, we first performed experimental studies where human participants performed an iso‐time constant‐power plantar flexion type of exercise to task failure and collected muscle activation, intramuscular metabolite level and muscle fascicle length data before and after the exercise. Then, we developed a human skeletal muscle model that accounts for both the cross‐bridge cycle and the associated metabolic processes, such as ATPase (myosin‐associated), creatine kinase, adenylate kinase, glycolysis and pH buffering. We tailored the model to the dynamic planter flexion exercise, used the muscle activation (from EMG) and fascicle length data as inputs, and parameterized the model to simulate sustained force generation and alterations in intramuscular metabolite levels as outputs, representing an average of five subjects. Subsequently, we used experimental data for the same parameters collected from two additional subjects to validate the model results. Our model was able to successfully predict the muscle force generation and metabolite alterations within the observed root mean square error between the experiments. Following this, we deployed the model to characterize the effect of different factors on force generation. We first investigated the relationship between the observed muscle activation pattern and force generation and related it to the accumulation of metabolites. We then evaluated the individual effect of metabolites, that is, ADP, P_i_ and H^+^, on force generation capacity to quantify their contribution to force inhibition and muscle fatigue.

## 2 METHODS

### 2.1 Subjects

We enrolled 11 recreationally active subjects (six males, five females; age, 23 ± 2 years; height, 172 ± 9 cm; weight, 68 ± 14 kg) and obtained their written informed consent to participate in this study. Two participants (one male, one female) withdrew from the protocol, and the data for two participants (two males) could not be included in the ^31^P‐MRS analysis due to poor signal‐to‐noise ratio or inconsistencies in the estimation of critical power. All participants were non‐smokers; were free of diabetes and had no known cardiovascular, neuromuscular or pulmonary disease; and were not taking any medications that affect muscle function. Using a questionnaire and accelerometry (GT3X, Actigraph, Pensacola, FL, USA), instrumented on the non‐dominant wrist for 7–10 days, we confirmed that the participants did not engage in any structured physical activity more than three times a week. Prior to the start of the protocol, we familiarized the participants with all the testing procedures. Participants fasted overnight, and all experimental trials were conducted in a thermoneutral environment at the University of Massachusetts Amherst. The study conformed to the Declaration of Helsinki and the Institutional Review Board at the University of Massachusetts and the Office of Human Research Oversight at Fort Detrick, MD, approved the study protocol.

### 2.2 Iso‐time constant‐power plantar flexion exercise protocol

#### 2.2.1 Exercise set‐up

**FIGURE 1:**
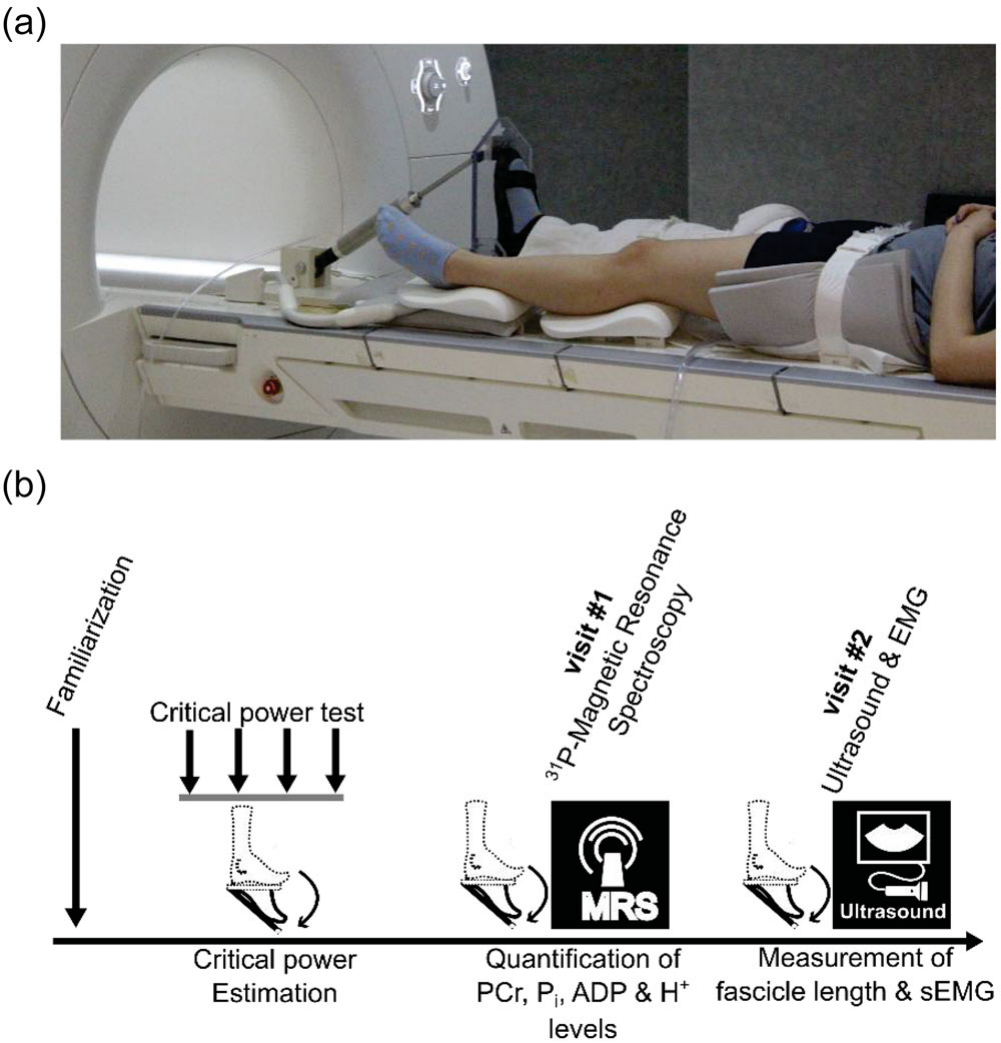
Schematic illustration of the plantar flexion exercise protocol used in this study. (a) Participants, in a supine position, performed plantar flexion movement on a custom ergometer consisting of a foot pedal connected to a non‐ferromagnetic cylinder piston. The pressure inside the cylinder is controlled using an air compressor. The ergometer is compact enough to be placed inside an MRI scanner (Skyra, Siemens). (b) Schematic outline of the different sessions that were part of the constant‐power plantar flexion experiment conducted in this study. Subjects completed a familiarization session, after which we estimated the critical power (CP) and work rate (*W*′) required to reach muscle failure within 10 min based on four trials, carried out at 48‐h intervals. Next, the subjects visited the laboratory for two separate sessions where they performed iso‐time constant‐power plantar flexion exercise at a work rate of *W*′ for ∼10 min. During their first of these visits, we measured the concentration of ADP, P_i_, H^+^ and phosphocreatine in the skeletal muscles of the actively exercising subjects using ^31^P‐magnetic resonance spectroscopy. During the second visit, we recorded the surface electromyography (sEMG) of the actively exercising subjects using bipolar surface electrodes. During this visit, we also measured the changes in fascicle length and pennation angle associated with plantar flexion motion in these subjects.

Participants performed dynamic (0.75 Hz) plantar flexion exercise to fatigue while lying supine using a custom‐designed ergometer with a constant resistance controlled by a double‐acting air cylinder (McMaster‐Carr, Elmhurst, IL, USA) (Figure 1a). Briefly, the ergometer consisted of a foot pedal, with a range of motion of ∼15°–20°, attached to a non‐ferromagnetic cylinder piston that was in turn connected to an air compressor. The air compressor was used to modulate the resistance experienced by the participants while performing plantar flexion motion on the foot pedal. We measured the range of motion using a potentiometer (model 3590; Bourns, Riverside, CA, USA) mounted on the pedal and interfaced with an MP160 analog‐to‐digital converter (Biopac Systems, Goleta, CA, USA). Our custom ergometer design was compact enough to fit into the bed of a Siemens Skyra scanner (Siemens Medical Solutions, Erlangen, Germany). Since the plantar flexion cycles were executed successively without break, half of each cycle was spent in active plantar flexion and the other half in relaxation, implying a duty cycle of ∼50%.

#### 2.2.2 Estimation of critical power and work rate for iso‐time constant‐power plantar flexion exercise

Before participants performed the iso‐time constant‐power plantar flexion exercise, we estimated the critical power (CP) and the maximum amount of work that can be performed above CP (*W*′) for each subject, from four trials. We defined task failure as the inability to successfully maintain the full range of motion (<15% of initial displacement) for three consecutive contraction cycles. We repeated this calculation for different work rates (*P*) randomized within the range of ∼4–9 W to obtain a range of task failure times (*t*), varying between 1 and 15 min. Each of the above visits was separated by 48 h and was preceded by a 3‐min warmup and followed by a 5‐min recovery period. We estimated CP and *W*′ by fitting the equation *P* = *W*′(1/*t*) + CP against the *P versus* 1/*t* curve obtained from the above trials (Broxterman et al., 2017). We then used the same equation alongside the estimated CP and *W*′ to calculate the work rate, *P*_10_ (W), needed to reach task failure within 10 min.

#### 2.2.3 Iso‐time constant‐power plantar flexion exercise

Once the CP was estimated during the initial visits, each participant then performed a constant‐power plantar flexion exercise, with the targeted power set to reach fatigue within 10 min and the exact time to fatigue recorded so that subsequent visits were of similar duration (Figure 1b). During each participant's first visit, we quantified the concentration of metabolites, such as ATP, ADP, P_i_, H^+^ and PCr, in the muscles during the course of the exercise using ^31^P‐MRS. During the participant's second visit to the laboratory, using bipolar surface electrodes, we measured EMG changes in the lower legs, including the gastrocnemius medialis, gastrocnemius lateralis, tibialis anterior and vastus lateralis, during the course of an iso‐time exercise. In addition, we measured the pennation angle and fascicle length of the medial and lateral gastrocnemius muscles using an ultrasound (Brennan et al., 2017; Loram et al., 2006), before and after exercise.

### 2.3 Measurement protocols

#### 2.3.1 ^31^P‐MRS

We performed ^31^P‐MRS using a whole‐body 3 T MRI system (Siemens Skyra) operating at 49.9 MHz for ^31^P resonance and running on the VE11C platform. We acquired the ^31^P‐MRS data using a dual‐tuned ^31^P‐proton (^1^H) custom‐made surface coil with linear polarization positioned under the gastrocnemius muscle. The ^31^P single‐loop coil diameter was 80 mm, which was surrounded by a 100‐mm ^1^H coil loop (Stark Contrast, Erlangen, Germany). After a three‐plane proton image to determine the position of the leg with respect to the surface coil, we performed advanced localized volume shimming followed by manual shimming (full width at half maximum < ^1^H: 45 Hz). Before each experiment, we acquired two fully relaxed spectra at rest with five averages per spectrum and a repetition time of 30 s. Then, we performed MRS data acquisition throughout the rest–exercise–recovery protocol using a free‐induction‐decay pulse sequence with a 0.1‐ms excitation radiofrequency rectangular pulse and the following parameters: repetition time of 2 s, receiver bandwidth of 4 kHz, 1024 data points, and five averages per spectrum. Supporting information Figure S1 shows a slack plot of the ^31^P spectra obtained for one of the participants. We quantified the saturation factors by comparing fully relaxed (repetition time = 30 s) and partially relaxed (repetition time = 2 s) spectra.

As previously described (Layec et al., 2008), we obtained the [PCr], [P_i_] and [ATP] using a time‐domain fitting routine using the AMARES algorithm (Vanhamme et al., 1997) incorporated into CSIAPO software. We calculated the intracellular pH from the chemical shift difference between the P_i_ and PCr signals. We calculated the free cytosolic [ADP] from [PCr] and pH using the creatine kinase equilibrium constant (*K*_CK_ = 1.66 × 10^9^ M^−1^) and assuming that PCr represents 85% of the total creatine content (Jeneson et al., 1996). The concentration of the H_2_PO_4_^−^ was calculated as:

(1) $$H_{2}{\text{PO}}_{4}-=\left[P_{i}\right]/\left(1+10^{\mathrm{pH}-6.75}\right)$$

We calculated the free cytosolic adenosine monophosphate (AMP) based on the equilibrium of the adenylate kinase reaction corrected for the effects of pH, assuming a free magnesium concentration of 1 mM. We calculated the resting concentrations from the average peak areas of the two relaxed spectra recorded at rest and assuming an 8.2 mM [ATP] (Harris et al., 1974) under these conditions.

#### 2.3.2 Surface EMG measurements

We used bipolar surface electrodes to measure EMG changes occurring in the medial and lateral gastrocnemius muscles during the plantar flexion exercise to task failure (iso‐time with first visit). We pre‐amplified (gain 1000) and filtered (common mode rejection ratio 20–500 Hz) the signal using a commercialized system (model MP160; Biopac Systems). We sampled the analog signal at a rate of 2000 Hz. We normalized the time‐dependent changes in integrated EMG (iEMG) obtained from the raw EMG signal with peak EMG signal from three 5‐s isometric maximal voluntary contractions and averaged them over 10 s. For modelling purposes, we converted the normalized EMG data from time scale to plantar flexion cycles using the average number of cycles executed by a subject in a 10 s duration (6.3 cycles). We used the average of the normalized gastrocnemius medialis and gastrocnemius lateralis EMG signals as the model input.

#### 2.3.3 Muscle geometry

We determined the pennation angle (PA) of the medial and lateral gastrocnemius muscles from images taken along the longitudinal axis of the muscle belly at rest utilizing a two‐dimensional, B‐mode ultrasound (12‐MHz probe) (Logiq P9; GE Healthcare, Chicago, IL, USA) (Brennan et al., 2017). We chose the measurement point corresponding to 30% of the shank length (measured as lateral tibial condyle to lateral malleolus) distal from the medial tibial condyle along the muscle belly. For the fascicle length (FL), we placed transducers at ∼30% of the shank length. We used the following formula to calculate FL:

(2) $$\mathrm{FL}=\mathrm{visible}\;\mathrm{fascicle}\;\mathrm{length}+h/\sin\left(\mathrm{PA}\right)$$

where *h* represents the vertical distance between the intersection of the visible fascicle with the image border and the deep aponeurosis, and PA denotes the pennation angle of the tracked fascicle. We repeated the measurements at two different angles, replicating the range of motion during the plantar flexion exercise (90° and ∼35–40° range of motion/7 cm displacement) before and after task failure.

#### 2.3.4 Calculation of sarcomere shortening velocity

We calculated the sarcomere shortening velocity as follows

(3) $$\frac{dL}{dt}=\frac{\Delta \mathrm{FL}}{N^{\mathrm{Sar}}\times T^{\mathrm{cyc}}}\;$$

where ΔFL represents the change in fascicle length for a 7‐cm displacement in plantar flexion motion, which is the difference between fascicle lengths measured with the feet positioned at 90° versus at 30–40° (corresponding to a 7‐cm displacement in plantar flexion) relative to the lower leg, *N* ^Sar^ (= 17,600; Huijing, 1985) denotes the number of sarcomeres, and *T* ^cyc^ represents the time taken for a plantar flexion cycle by the subject.

### 2.4 Model development and simulations

#### 2.4.1 Modelling cross‐bridge cycling and force generation

We used the five‐state model (illustrated in Figure 2a) of Tewari et al. (2016) to simulate cross‐bridge kinetics and force generation. We used *N*(*t*), *P*(*t*), *p*_1_(*t*, *s*), *p*_2_(*t*, *s*) and *p*_3_(*t*, *s*) to represent state probabilities of the five states (N, P, A_1_, A_2_ and A_3_), where *s* denotes the strain on the attached states (A_1_, A_2_ and A_3_). We evaluated the fraction of total cross‐bridges in one of the three attached states, at time *t*, by $\hat{p_{i}}\;\left(t\right)=\int_{-\infty }^{\infty }p_{i}\left(t,s\right)ds\;$. The mean strain of the cross‐bridges in one of the three attached states is given by $\int_{-\infty }^{+\infty }sp_{i}\left(t,s\right)ds/\hat{p_{i}}\left(t\right)$. Since the total number of actin–myosin complexes does not change with time at any given time, the sum of all fractional state probabilities (both unbound and bound cross‐bridge states) equals 1.

(4) $$N\left(t\right)+P\left(t\right)+\int_{-\infty }^{+\infty }p_{1}\left(t,s\right)ds+\int_{-\infty }^{+\infty }p_{2}\left(t,s\right)ds+\;\int_{-\infty }^{+\infty }p_{3}\left(t,s\right)ds=1$$

**FIGURE 2:**
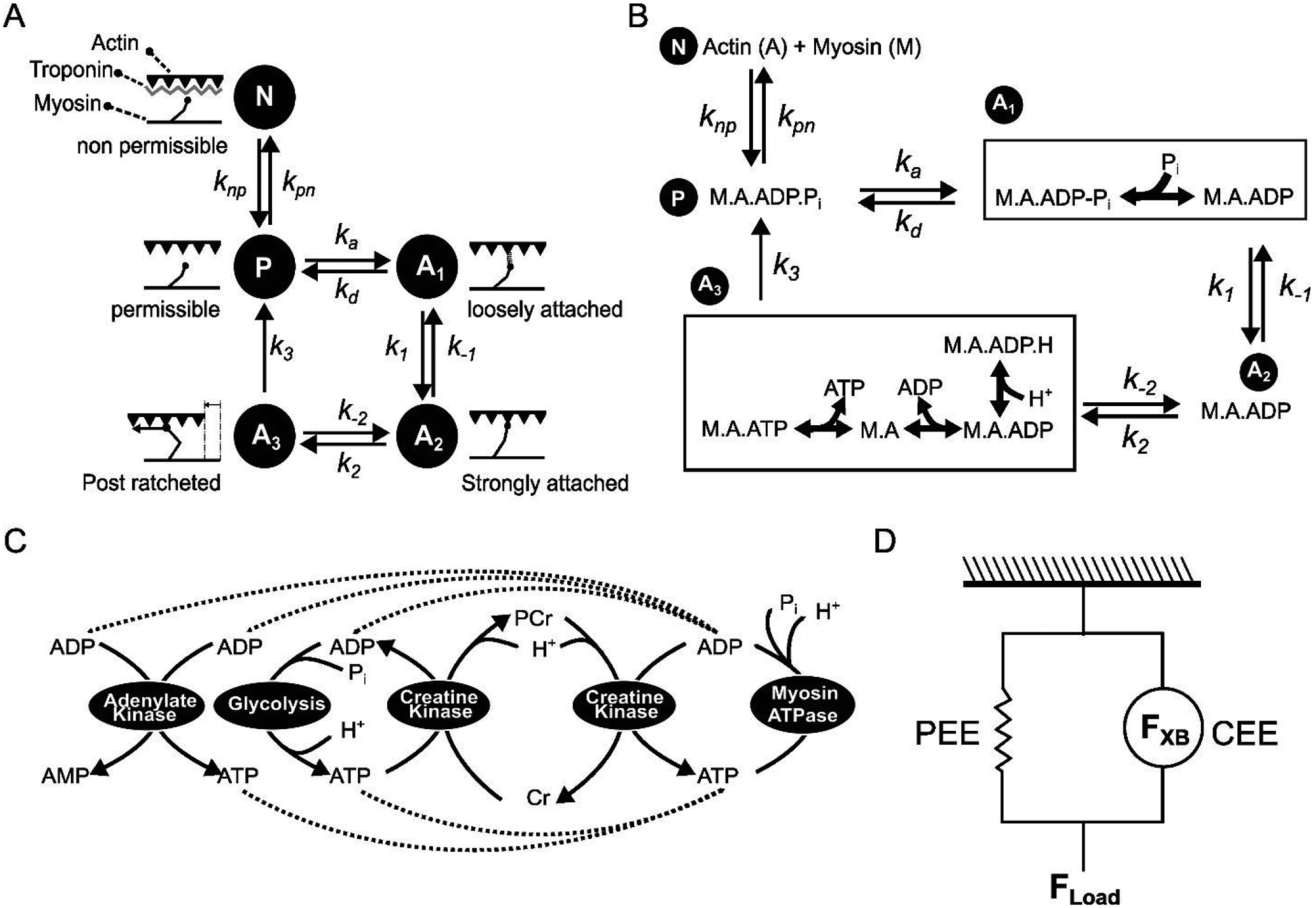
Schematic illustration of a cross‐bridge cycling model for skeletal muscle force generation. (a) The 5‐state model of the actin–myosin cross‐bridge cycle (Tewari et al., 2016). In the non‐permissible state (N), the binding sites on the actin filament are blocked by troponin, preventing any interaction with myosin. The permissible state (P) represents the state in which actin binding sites are free of troponin and open for myosin interaction. A_1_ represents the state in which actin and myosin are loosely bound. A_2_ represents the pre‐power‐stroke state where actin and myosin are strongly bound. A_3_ is the post‐power‐stroke state where actin and myosin are still strongly bound. The rate constants *k*_np_, *k*_pn_, *k*_a_, *k*_d_, *k*_1_, *k*_−1_, *k*_2_, *k*_−2_ and *k*_3_ model the transitions between these states. (b) The rapid equilibrium transition steps involved in the cross‐bridge cycling model. Transitions between some of these states (indicated by a box) require the association/dissociation of metabolites, such as ATP, ADP, H^+^ and P_i_. Accordingly, the rate constants of these transitions are modelled to account for the appropriate dissociation constants representing these interactions as binding polynomials. (c) Schematic illustration of different metabolic processes included in the model that provide the ATP needed for cross‐bridge cycling. (d) Schematic illustration of overall skeletal muscle force generation. Here, the total force (*F*_Load_) is the sum of the force (*F*_XB_) generated by the contractile element (CEE) and the parallel elastic elements (PEE).

The equations that govern the kinetics of cross‐bridge cycling and its metabolite‐mediated modulations are depicted as follows:

(5) $$\frac{dN}{dt}=-k_{\mathrm{np}}N\left(t\right)+k_{\mathrm{pn}}P\left(t\right)$$

(6) $$\frac{dP}{dt}=k_{\mathrm{np}}N\left(t\right)-k_{\mathrm{pn}}P\left(t\right)+{\tilde{k}}_{d}p_{1}\left(t,s\right)-k_{a}P\left(t\right)+{\tilde{k}}_{3}e^{\alpha_{3}{\left(s+s_{3}\right)}^{2}}p_{3}\left(t,s\right)$$

(7) $$\frac{\partial p_{1}}{\partial t}+\frac{dL}{dt}\frac{\partial p_{1}}{\partial s}=k_{a}\delta \left(s\right)P\left(t\right)-{\tilde{k}}_{d}p_{1}-{\tilde{k}}_{1}e^{-\alpha_{1}s}p_{1}+{\tilde{k}}_{-1}e^{+\alpha_{1}s}p_{2}$$

(8) $$\frac{\partial p_{2}}{\partial t}+\frac{dL}{dt}\frac{\partial p_{2}}{\partial s}={\tilde{k}}_{1}e^{-\alpha_{1}s}p_{1}-k_{-1}e^{+\alpha_{1}s}p_{2}-k_{2}e^{-\alpha_{2}s}p_{2}+{\tilde{k}}_{-2}p_{3}$$

(9) $$\frac{\partial p_{3}}{\partial t}+\frac{dL}{dt}\frac{\partial p_{3}}{\partial s}=k_{2}e^{-\alpha_{2}s}p_{2}-{\tilde{k}}_{-2}p_{3}-{\tilde{k}}_{3}e^{\alpha_{3}{\left(s+s_{3}\right)}^{2}}p_{3}$$

(10) $$k_{\mathrm{np}}=\beta \times \mathrm{iEM}G_{j}$$

(11) $$k_{\mathrm{pn}}=\beta \times \left(1-\mathrm{iEM}G_{j}\right)$$

(12) $${\tilde{k}}_{d}=k_{d}\frac{\left[P_{i}\right]/K_{\mathrm{Pi}}}{1+\left[P_{i}\right]/K_{\mathrm{Pi}}}$$

(13) $${\tilde{k}}_{1}=k_{1}\frac{1}{1+\left[P_{i}\right]/K_{\mathrm{Pi}}}$$

(14) $${\tilde{k}}_{-2}=k_{-2}\frac{\left[\mathrm{ADP}\right]/K_{\mathrm{ADP}}}{1+\left[\mathrm{ADP}\right]/K_{\mathrm{ADP}}+\left[\mathrm{ATP}\right]/K_{\mathrm{ATP}}+\left(\left[\mathrm{ADP}\right]/K_{\mathrm{ADP}}\times \left[H^{+}\right]/K_{H+}\right)}$$

(15) $${\tilde{k}}_{3}=k_{3}\frac{\left[\mathrm{ATP}\right]/K_{\mathrm{ATP}}}{1+\left[\mathrm{ADP}\right]/K_{\mathrm{ADP}}+\left[\mathrm{ATP}\right]/K_{\mathrm{ATP}}+\left(\left[\mathrm{ADP}\right]/K_{\mathrm{ADP}}\times \left[H^{+}\right]/K_{H+}\right)}$$

A complete description of all the parameters used in Equations (4)–(15) is presented in Table 1. Equations (10) and (11) mimic the overall muscle activation captured by the EMG data. iEMG_j_ denotes the normalized EMG data for cycle j calculated as described in Section 2.3.2, *L* represents sarcomere length, d*L*/d*t* denotes sarcomere shortening velocity, and [P_i_], [ADP], [ATP] and [H^+^] represent the concentrations of P_i_, ADP, ATP and H^+^, respectively. We modified Equations (14) and (15) from Tewari et al. (2016)) to account for proton‐mediated modulation of the state A_3_→P and A_3_→A_2_ transitions (as depicted in Figure 2b) based on the mechanisms proposed in Jarvis et al. (2018) for H^+^‐mediated inhibition of cross‐bridge kinetics.

**TABLE 1:** Estimates of model parameters from human subject data (*n* = 5) compared to mouse and rat values compiled from the literature.

Parameter	Description	Human	Mouse*	Rat*	Units
Cross‐bridge parameters					
*k*_a_	Rate of actin–myosin transition from permissible state to loosely attached state	3.6	454 (3.3)	294 (18)	s^−1^
*k*_d_	Rate of actin–myosin transition from loosely attached state to permissible state	39.7	1.25 (3.7)	35.5 (3)	s^−1^
*k*_1_	Rate of cross‐bridge transition from loosely bound to strongly bound state	13.4	41.2 (0.9)	10.2 (0.1)	s^−1^
*k*_−1_	Rate of cross‐bridge transition from strongly bound to loosely bound state	11.8	17.6 (0.1)	10.3 (0.8)	s^−1^
*k*_2_	Rate of ratcheting	16.7	159.3 (12.9)	88.6 (3.8)	s^−1^
*k*_−2_	Rate of unratcheting	18.9	153.7 (0.04)	2.1 (0.2)	s^−1^
*k*_3_	Rate of actin–myosin detachment	18.7	87.7 (5.3)	35.6 (0.8)	s^−1^
α_1_	Stretch sensing parameter for *k*_1_ and *k*_−1_	36.6	15.1 (0.01)	10 (0.1)	µm^−1^
α_2_	Stretch sensing parameter for *k*_2_	171.8	10.1 (0.04)	9.1 (0.9)	µm^−1^
α_3_	Stretch sensing parameter for *k*_3_	80.1	50.2 (0.3)	59.3 (0.9)	µm^−1^
*s*_3_	Stretch in state A_3_ at which *k*_3_ is minimum	2.0	9.9 (0.1)	9.9 (0.1)	nm
*K*_ATP_	ATP dissociation constant	5,956.2	597 (55)	489 (13.2)	µM
*K*_ADP_	ADP dissociation constant	0.06	0.194	0.194	mM
*K*_Pi_	P_i_ dissociation constant	21.2	4 (0.1)	4	mM
*K*_H+_	H^+^ dissociation constant	0.292	NA	NA	µM
Force generation parameters					
*k*_stiff,1_	Stiffness constant of frictional forces during actin–myosin interaction	87,091.4	1137 (4)	2,827.1 (54)	mN mm^−2^ µm^−1^
*k*_stiff,2_	Stiffness constant of forces generated during the cross‐bridge power stroke	28,173	19,066 (11)	51,871 (526)	mN mm^−2^ µm^−1^
Metabolic parameters					
*k*_CKf_	Rate constant for creatine kinase (ATP forming)	0.43	NA	NA	mM^−1^ s^−1^
*k*_CKr_	Rate constant for creatine kinase (ADP forming)	0.0026	NA	NA	mM^−1^ s^−1^
*k*_Gly_	Rate constant for glycolysis	0.26	NA	NA	mM^−1^ s^−1^
*k*_Pi,dil_	Rate constant for P_i_ dilution or export from myocytes	0.004	NA	NA	s^−1^
*k*_adk_	Rate constant for adenylate kinase	28.3	NA	NA	mM^−1^ s^−1^

Values in parentheses are standard deviations. *Data from Tewari et al. (2016). NA, not applicable.

#### 2.4.2 Calculation of force generated from cross‐bridge cycling

In the 5‐state model, the force, *F*_XB_, generated due to cross‐bridge cycling and intermittent attachment–detachment of actin–myosin filaments is given by the following equation:

(16) $$F_{\mathrm{XB}}=k_{\mathrm{stiff},1}\;\left(\underset{-\infty }{\int^{+\infty }}sp_{2}\left(t,s\right)ds+\underset{-\infty }{\int^{+\infty }}sp_{3}\left(t,s\right)ds\right)+k_{\mathrm{stiff},2}\Delta_{r}\underset{-\infty }{\int^{+\infty }}p_{3}\left(t,s\right)ds$$

where *k*_stiff,1_ represents the stiffness of frictional forces arising due to actin–myosin interaction, *k*_stiff,2_ denotes the stiffness of ratcheted cross‐bridges, and $\Delta_{r}$ represents the power stroke size. The total force, *F*_total_, produced by the cross‐bridge model was then calculated as follows:

(17) $$F_{\mathrm{total}}=F_{\mathrm{XB}}+F_{\mathrm{PEE}}$$

We calculated the force accounting for parallel elastic elements, *F*_PEE_, using the method of Rockenfeller et al. (2020).

#### 2.4.3 Modelling the dynamics of muscle metabolite alterations

We found in the literature that changes in ATP, ADP, P_i_, H^+^ and PCr levels are the result of coordinated activities of the following metabolic processes: (1) myosin‐associated ATP hydrolysis (Lymn & Taylor, 1971), (2) creatine kinase (Meyer et al., 1984; Paul, 1983), (3) glycolysis (Gastin, 2001), and (4) adenylate kinase (Janssen et al., 2003), as illustrated in Figure 2c. Table 2 lists the stoichiometry of these reactions and the rate expressions used to model their contributions. During each cross‐bridge cycle, ATP binds to myosin and becomes hydrolysed, releasing ADP, P_i_ and H^+^ (Lymn & Taylor, 1971). This ATP hydrolysis, which immediately follows the power stroke, leads to the detachment of myosin from the actin binding site, which frees up myosin to bind to a new actin site, leading to the next cross‐bridge cycle. In our five‐state cross‐bridge model, ATP binds to myosin at the A_3_ state and is hydrolysed during the A_3_→P transition. Given that 1 mole of ATP is hydrolysed for each cross‐bridge cycle, the rate of ATP hydrolysis equals the rate of transition from A_3_ to P, which is in turn a function of *k*_3_, *s*, and *p*_3_(*t*, *s*). So, we used the rate expression for the A_3_→P transition to model the ATP hydrolysis processes accompanying muscle contraction.

**TABLE 2:** Metabolic processes, their stoichiometric chemical equations and rate expressions used in our model.

No.	Reaction/pathway	Chemical equation	Rate formulation
1	ATP hydrolysis	^*^ $\mathrm{ATP}+H_{2}O\to \mathrm{ADP}+P_{i}+0.6H^{+}$	${\tilde{k}}_{3}e^{\alpha_{3}{\left(s+s_{3}\right)}^{2}}p_{3}$
2	Creatine kinase (ATP buffering)	$\mathrm{PCr}\;+\;\mathrm{ADP}\;+H^{+}\to \;\mathrm{ATP}\;+\;\mathrm{Cr}$	$k_{\mathrm{CKf}}\left[\mathrm{PCr}\right]\left[\mathrm{ADP}\right]$
3	Creatine kinase (PCr regenerating)	$\mathrm{ATP}\;+\;\mathrm{Cr}\;\to \;\mathrm{PCr}\;+\;\mathrm{ADP}\;+H^{+}$	$k_{\mathrm{CKr}}\left({\left[\mathrm{PCr}\right]}_{0}-\left[\mathrm{PCr}\right]\right)\left[\mathrm{ADP}\right]$
4	Glycolysis	$\mathrm{Glucose}\;+2\mathrm{NA}D^{+}+2\mathrm{ADP}\;+2P_{i}\to 2\mathrm{Pyruvate}\;+2\mathrm{NADH}\;+2H^{+}+2\mathrm{ATP}\;+2H_{2}O$	$k_{\mathrm{Gly}}\left[\mathrm{ADP}\right]\left[P_{i}\right]$
5	Adenylate kinase	ADP+ADP→ATP+AMP	$k_{\mathrm{adk}}\left[\mathrm{ADP}\right]\left[\mathrm{ADP}\right]$

Cr, creatine; PCr, phosphocreatine. *The stoichiometric coefficient of H^+^ was set to 0.6 based on the ratio in which H_2_PO_4_^−^ and HPO_4_^2−^ constitute P_i_ at physiological pH (Kushmerick, 2011).

We accounted for both the ATP‐buffering and PCr‐regenerating activities of creatine kinase. We modelled the rate expression for ATP‐buffering activity as a function of [ADP] and [PCr], assuming second‐order rate kinetics. Similarly, we modelled the rate expression for PCr‐regenerating activity as a function of [ATP] and [creatine], assuming second‐order rate kinetics. Because we did not measure the creatine level during the experimental exercise, we indirectly estimated it by subtracting the [PCr] at any given time from the concentration estimated at the beginning of the exercise ([PCr]_0_). We modelled the rate of glycolytic ATP synthesis using second‐order rate kinetics with respect to [ADP] and [P_i_]. Similarly, we modelled the rate of adenylate kinase reaction as a function of [ADP] assuming second‐order rate kinetics. We also accounted for the buffering capacity of P_i_ using its buffering capacity (γ) as detailed in Kemp et al. (1993). Considering all the above processes, we modelled the dynamics of ATP, ADP, PCr, P_i_ and H^+^ using the following equations:

(18) $$\frac{d[P_{i}]}{dt}={\tilde{k}}_{3}e^{\alpha_{3}{\left(s+s_{3}\right)}^{2}}p_{3}-k_{\mathrm{Gly}}\left[\mathrm{ADP}\right][P_{i}]-k_{\mathrm{Pi},\mathrm{dil}}[P_{i}]$$

(19) $$\begin{array}{ccc} \frac{d\left[H^{+}\right]}{dt} & = & 0.6\times {\tilde{k}}_{3}e^{\alpha_{3}{\left(s+s_{3}\right)}^{2}}p_{3}+k_{\mathrm{Gly}}\left[\mathrm{ADP}\right]\left[P_{i}\right]-k_{\mathrm{CKf}}\left[\mathrm{PCr}\right]\left[\mathrm{ADP}\right]\\ & & +\;k_{\mathrm{CKr}}\left({\left[\mathrm{PCr}\right]}_{0}-\left[\mathrm{PCr}\right]\right)\left[\mathrm{ADP}\right]+\gamma \Delta \mathrm{pH} \end{array}$$

(20) $$\begin{array}{ccc} \frac{d\left[\mathrm{ADP}\right]}{dt} & = & {\tilde{k}}_{3}e^{\alpha_{3}{\left(s+s_{3}\right)}^{2}}p_{3}-k_{\mathrm{Gly}}\left[\mathrm{ADP}\right]\left[P_{i}\right]-k_{\mathrm{CKf}}\left[\mathrm{PCr}\right]\left[\mathrm{ADP}\right]\\ & & +\;k_{\mathrm{CKr}}\left({\left[\mathrm{PCr}\right]}_{0}-\left[\mathrm{PCr}\right]\right)\left[\mathrm{ADP}\right]-k_{\mathrm{adk}}\left[\mathrm{ADP}\right]\left[\mathrm{ADP}\right] \end{array}$$

(21) $$\frac{d\left[\mathrm{PCr}\right]}{dt}=]-k_{\mathrm{CKf}}\left[\mathrm{PCr}\right]\left[\mathrm{ADP}\right]+k_{\mathrm{CKr}}\left({\left[\mathrm{PCr}\right]}_{0}-\left[\mathrm{PCr}\right]\right)\left[\mathrm{ADP}\right]$$

(22) $$\frac{d\left[\mathrm{ADP}\right]}{dt}=-\frac{d\left[\mathrm{ATP}\right]}{dt}$$

Table 1 contains a complete description of all the parameters used in Equations (18)–(22).

#### 2.4.4 Simulation of plantar flexion cycles

For each plantar flexion cycle, we solved Equations 4–22 simultaneously, for a cycle period of 1.6 s, to evaluate the time‐dependent fractions of the N, P, A_1_, A_2_ and A_3_ states and the levels of ATP, ADP, PCr, P_i_ and H^+^. We calculated the force generated using Equation 17. For the first plantar flexion cycle, we set the fractions of the P, A_1_, A_2_ and A_3_ states to 0 and the fraction of the N state to 1. For subsequent cycles, we initiated the A_1_, A_2_ and A_3_ states from 0 and used the following equation to initiate the P state:

(23) $$P{\left(t_{0}\right)}^{j}=\hat{p_{1}}{\left(t_{f}\right)}^{j-1}+\hat{p_{2}}{\left(t_{f}\right)}^{j-1}+\hat{p_{3}}{\left(t_{f}\right)}^{j-1}+P{\left(t_{f}\right)}^{j-1}$$

where j denotes the index of the current cycle, j−1 represents the index of the previous cycle, and *t*_0_ and *t*_f_ denote the beginning and end of the cycle (1.6 s), respectively. Similarly, for the first cycle, we initiated the ATP, ADP, PCr, P_i_ and H^+^ levels using those measured before exercise (average levels recorded for a period of 60 s with 10‐s sample intervals). For subsequent cycles, we used the final concentration values reached from the earlier cycle. We programmed and simulated the model in the MATLAB (R2022a) environment.

#### 2.4.5 Model parameterization

The model contains a total of 23 parameters for simulating the fractions of cross‐bridge states, metabolite levels and force generation. We estimated these parameters using a non‐linear least‐squares method by fitting the experimentally measured force, ADP, PCr and P_i_ levels for 240 plantar flexion cycles (*N*_c_). We used the averaged data from five of the seven participants for parameterization. We used the normalized EMG as input to the model and defined an objective function of non‐linear least squares for optimization as shown below:

(24) $$\begin{array}{ccc} f_{\mathrm{obj}} & = & \frac{\sum_{j=1}^{N_{c}}{\left({\left[P_{i}\right]}_{j}^{\mathrm{data}}-{\left[P_{i}\right]}_{j}^{\mathrm{Model}}\right)}^{2}}{\max({\left[P_{i}\right]}_{j}^{\mathrm{data}})}+\frac{\sum_{j=1}^{N_{c}}{\left({\left[\mathrm{ADP}\right]}_{j}^{\mathrm{data}}-{\left[\mathrm{ADP}\right]}_{j}^{\mathrm{Model}}\right)}^{2}}{\max({\left[\mathrm{ADP}\right]}_{j}^{\mathrm{data}})}\\ & & +\frac{\sum_{j=1}^{N_{c}}{\left({\left[\mathrm{PCr}\right]}_{j}^{\mathrm{data}}-{\left[\mathrm{PCr}\right]}_{j}^{\mathrm{Model}}\right)}^{2}}{\max({\left[\mathrm{PCr}\right]}_{j}^{\mathrm{data}})}+\frac{\sum_{j=1}^{N_{c}}{\left({F_{\mathrm{total}}}_{j}^{\mathrm{data}}-{F_{\mathrm{total}}}_{j}^{\mathrm{Model}}\right)}^{2}}{\max({F_{\mathrm{total}}}_{j}^{\mathrm{data}})} \end{array}$$

We used the MATLAB ‘fmincon’ function for the parameter estimation routine and repeated it 100 times starting from random points within the parameter space. We chose the best‐fit parameter set, which gave *f*_obj_ closest to zero, as the most accurate estimate and used it for the subsequent simulations.

#### 2.4.6 Sensitivity analysis

Once we obtained the parameters, we performed a sensitivity analysis to analyse the parameter space. To calculate the local sensitivities, we perturbed each parameter one at a time, by 1%, and evaluated the change in force relative to the change in parameter (Nagaraja et al., 2014; Wei et al., 2007). We determined the sensitivity values using the following equation:

(25) $$S_{i\left(x^{0}\right)}=\frac{\frac{F_{\mathrm{total}}\left(x_{1}^{0},\cdots ,x_{i}^{0}+dx_{i},\cdots ,x_{I}^{0}\right)-F_{\mathrm{total}}\left(x_{1}^{0},\cdots ,x_{i}^{0}-dx_{i},\cdots ,x_{I}^{0}\right)}{F_{\mathrm{total}}\left(x^{0}\right)}}{\left(2\times \frac{dx_{i}}{x_{i}^{0}}\right)}\;$$

where $x^{0}$ denotes to the best‐fit parameter set and $S_{i}$ represents the sensitivity with respect to force for the i^th^ parameter. For the global sensitivity analysis, we uniformly sampled 10,000 parameter sets in the vicinity of the optimal parameter set $x^{0}$, allowing for 10% variation in the value of each parameter (Nagaraja et al., 2014; Wei et al., 2007). We used Latin hypercube sampling (MATLAB function ‘lhsdesign’) to identify the 10,000 parameters sets. We then performed a local sensitivity analysis, as described above, for all 10,000 models defined by the sampled parameters. Finally, we used box plots to analyse the parameter sensitivities calculated with these models.

#### 2.4.7 Evaluating the effect of ADP, P_i_ and H^+^ levels

For each study, we simulated 240 plantar flexion cycles using the procedure described above. We evaluated the effect of each metabolite on force generation by simulating an additional 10% of the cycles (i.e., 24 cycles) with an increasing concentration of that metabolite, ranging from 1 to 2 times the concentration reached at the end of 240 cycles. During these additional cycles, we set the rate of change of concentration for the other metabolites to zero.

## 3 RESULTS

### 3.1 Experimental measurement of muscle activation and intramuscular metabolite alterations

**FIGURE 3:**
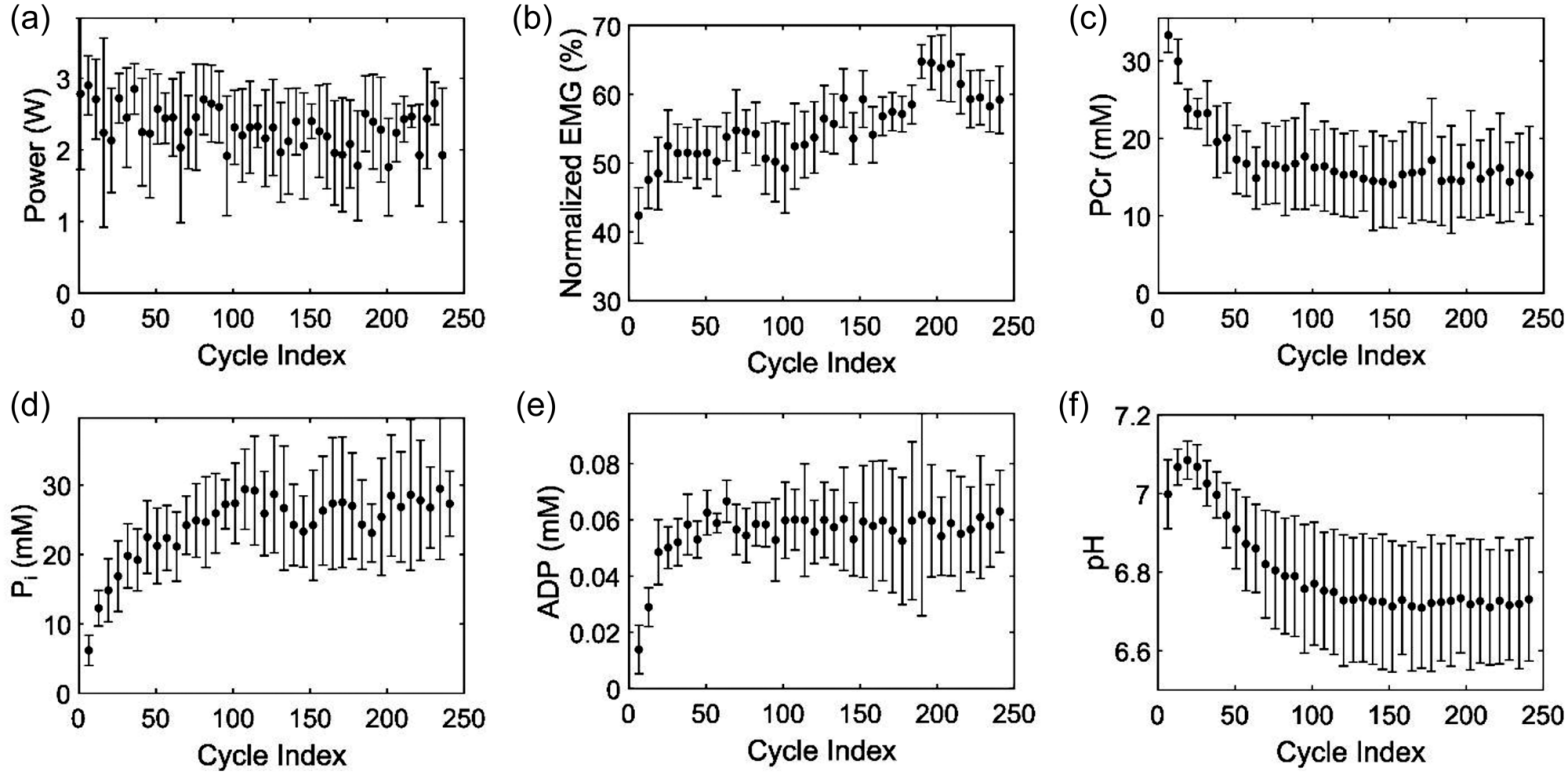
Experimental measurements during an iso‐time constant‐power dynamic plantar flexion exercise. (a) Average power generated as a function of plantar flexion cycles executed as part of the experiment. (b) Muscle activation measured as an average normalized integrated electromyogram (EMG) (%) as a function of plantar flexion cycles. (c–f) Average levels of intramuscular metabolite perturbations of phosphocreatine (PCr) (c), P_i_ (d), ADP (e), and H^+^ (f) as a function of plantar flexion cycles. Data are reported as an average of five subjects, and error bars indicate the experimental standard deviation between the subjects.

Figure 3 depicts the average power generated, the neuronal muscle activation required to sustain the power generation, and the evolution of metabolite profiles during the plantar flexion exercise (*n* = 5). As per the plantar flexion exercise protocol, all participants maintained a constant power, as depicted in Figure 3a. During the exercise, the power required to execute the plantar flexion cycles was constant (*P*_10_). However, to maintain this power, muscles either continuously recruited more motor units or increased their motor unit firing rate as shown in Figure 3b. Our measurement shows that the average iEMG signal increased monotonically from 36% to 60% as the exercise progressed. Furthermore, indicating energy expenditure, we observed a rapid decline in PCr levels within 100 s (60 cycles) and then a steady‐state level for the rest of the exercise (Figure 3c). In comparison, the levels of P_i_ (Figure 3d), ADP (Figure 3e), and H ^+^ (Figure 3f) increased rapidly at the beginning before reaching a plateau in the later part of the exercise. These observations were consistent with those reported in the literature for other types of exercise (Broxterman et al., 2017). Furthermore, our fascicle length measurements before and after participants performed a full range of plantar flexion motion indicated an average change of 1.1 µm (SD = 0.68; *n* = 4). Based on the average time taken to execute a plantar flexion cycle (∼1.6 s, *n* = 5), we calculated the average sarcomere shortening velocity as ∼0.68 µm/s for the five subjects (Equation 3).

### 3.2 The proposed skeletal muscle cross‐bridge model captures muscle force generation and metabolite alterations during a plantar flexion exercise

**FIGURE 4:**
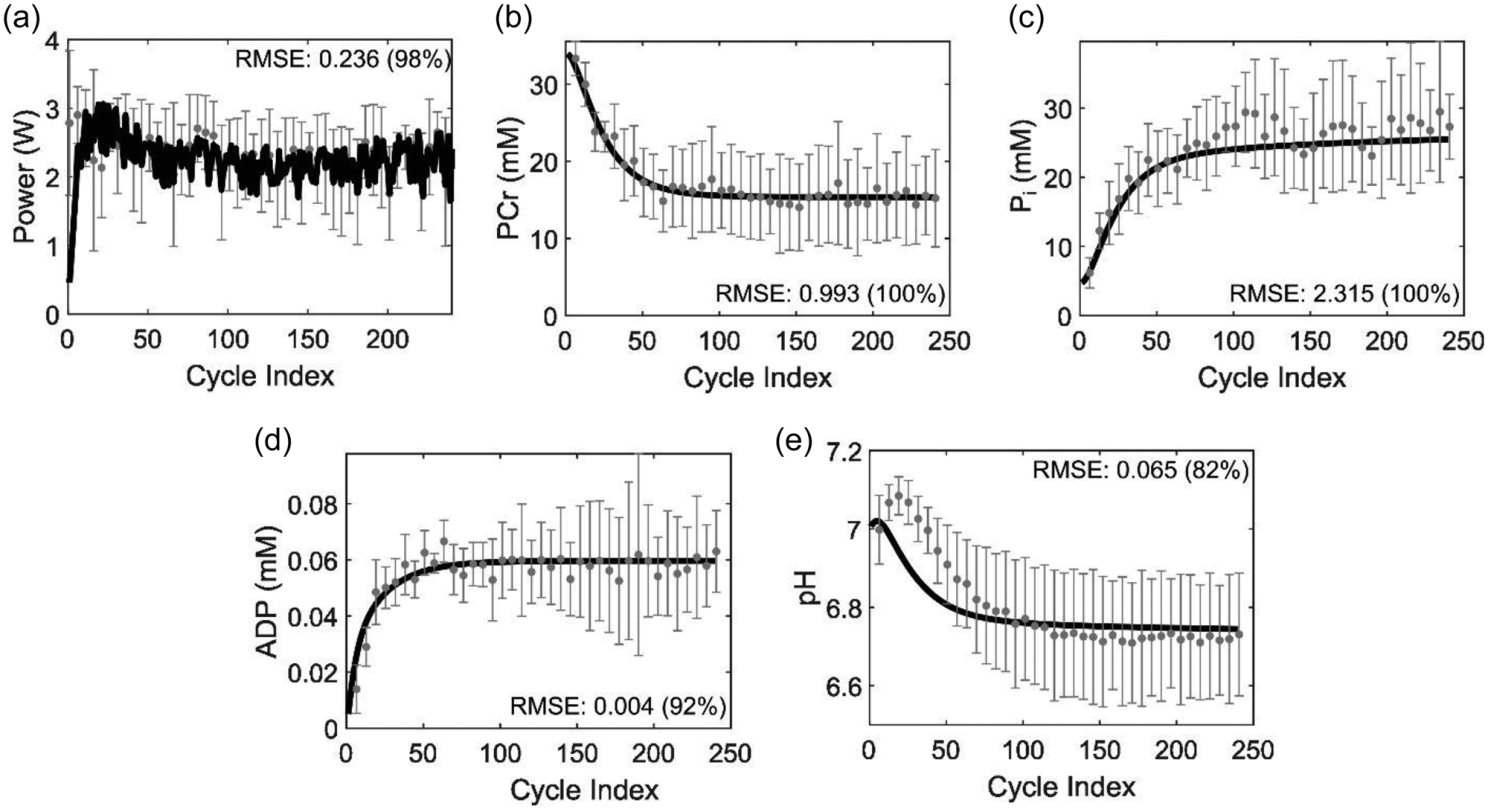
Cross‐bridge model simulation of dynamic plantar flexion exercise and parameterization. (a) Plot comparing model‐simulated power (continuous line) and experimentally recorded power (circles with error bar). (b–e) Model description of the intramuscular metabolite alterations (continuous lines) compared with the experimental data (circles with error bars) for phosphocreatine (PCr) (b), P_i_ (c), ADP (d), and H^+^ (e). Error bars indicate standard deviations (*n* = 5 subjects).

To accurately simulate the cross‐bridge cycles and their associated metabolite dynamics, we estimated 23 kinetic rate parameters using the normalized iEMG data and sarcomere shortening velocity as the model inputs and fitting the power, PCr, P_i_ and ADP profiles by minimizing the difference between the experimental data and the model simulations (Equation 24). We did not include the pH data in our parameterization process as we reserved it for model validation. Figure 4 shows a comparison of the model‐simulated (obtained with the optimal parameter set; continuous line) and experimental data (circles with error bars) for power, PCr, P_i_, ADP and pH. The initial increase in power, observed in our simulations (Figure 4a), is closely related to how the model is initiated for the first plantar flexion cycle. As described in Section 2.4.4, for the first cycle, the state probabilities of the unbound and bound states (P, A_1_, A_2_ and A_3_) were set to zero while the state probability of the non‐permissible state (N‐state) was set to 1. This follows our assumption that all the cross‐bridges are in the non‐permissible state before the start of exercise. As a result, it takes a few cycles before the model accumulates enough cross‐bridges in the P‐state to be able to produce the required force/power. Our skeletal muscle model was able to fit the experimental data with excellent accuracy across all four datasets. Table 3 summarizes the root mean square error (RMSE) values for different fits obtained as part of our parameterization process. We obtained a RMSE value of 0.24 W for power generation, with 98% of the simulated cycles generating the power within 1 standard deviation of the experimental data (Figure 4a). Similarly, the RMSE values for PCr (Figure 4b) and P_i_ (Figure 4c) were 1.0 and 2.3 mM, respectively, with the model fitting all the experimental data points within 1 standard deviation. The ADP fit had a RMSE of 4 µM, with the model fitting 97% of the experimental data points within 1 standard deviation (Figure 4d). Interestingly, our model was able to predict the alterations in pH (Figure 4e), the dataset we did not use as part of the optimization process, attesting to the accuracy of the model. The parameterized model was able to predict the measured pH dynamics with a RMSE of 0.065 pH units, with 82% of the predicted data points lying within 1 standard deviation of the experimentally measured values, providing a partial validation of the proposed skeletal muscle model.

**TABLE 3:** Root mean square error (RMSE) calculated between the model predictions and experimental datasets during the parameterization and validation steps.

No.	Data	RMSE between model simulations and experimental dataset in Figure 4, ^a^	RMSE between model predictions and experimental dataset in Figure 5, ^b^	RMSE between the two independent experimental datasets
1	Power, W	0.236 (98%)	0.600 (70.6%)	1.3
2	PCr, mM	0.993 (100%)	5.614 (50.8%)	8.5
3	P_i_, mM	2.315 (100%)	4.592 (41.3%)	7.0
4	ADP, mM	0.004 (92%)	0.014 (55.6%)	27.7
5	pH, pH units	0.065 (82%)	0.118 (60.3%)	0.2

Values in parentheses indicate the percentage of data points that were within one standard deviation of the experimental data points. PCr, phosphocreatine.

^a^ RMSE between the model predictions and the data used for parameterization, which were the average of the five subjects.

^b^ RMSE between the model predictions and the data used for validation, which was the average of the two additional subjects.

Table 1 shows the best‐fit parameter values obtained using our parameter estimation procedure. For 16 parameters, we were able to compare our estimates with those reported by Tewari et al. (2016) for mouse and rat cardiac muscle studies. Note that for certain parameters, for example, *k*_−2_, the mouse and rat estimates differed by more than one order of magnitude. For most of the 16 parameters, our estimates were on the same order of magnitude as those for either mouse or rat, and for only five parameters (*k*_a_, α_2_, *K*_ATP_, *K*_ADP_, and *k*_stiff,1_) did the estimates differ by an order of magnitude or more. We attribute these differences to various factors, including biological differences between species and muscle types as well as variations in the experimental conditions. Specifically, the experimental data used for estimating rat and mouse parameters were collected from myocardial strips excised from the left ventricle. In comparison, our data were collected from exercising human participants using non‐invasive techniques and account for the activities of different and diverse muscle types, such as the gastrocnemius medialis, gastrocnemius lateralis, tibialis anterior and vastus lateralis.

### 3.3 The proposed model predicts dynamic plantar flexion exercise from two independent subjects

**FIGURE 5:**
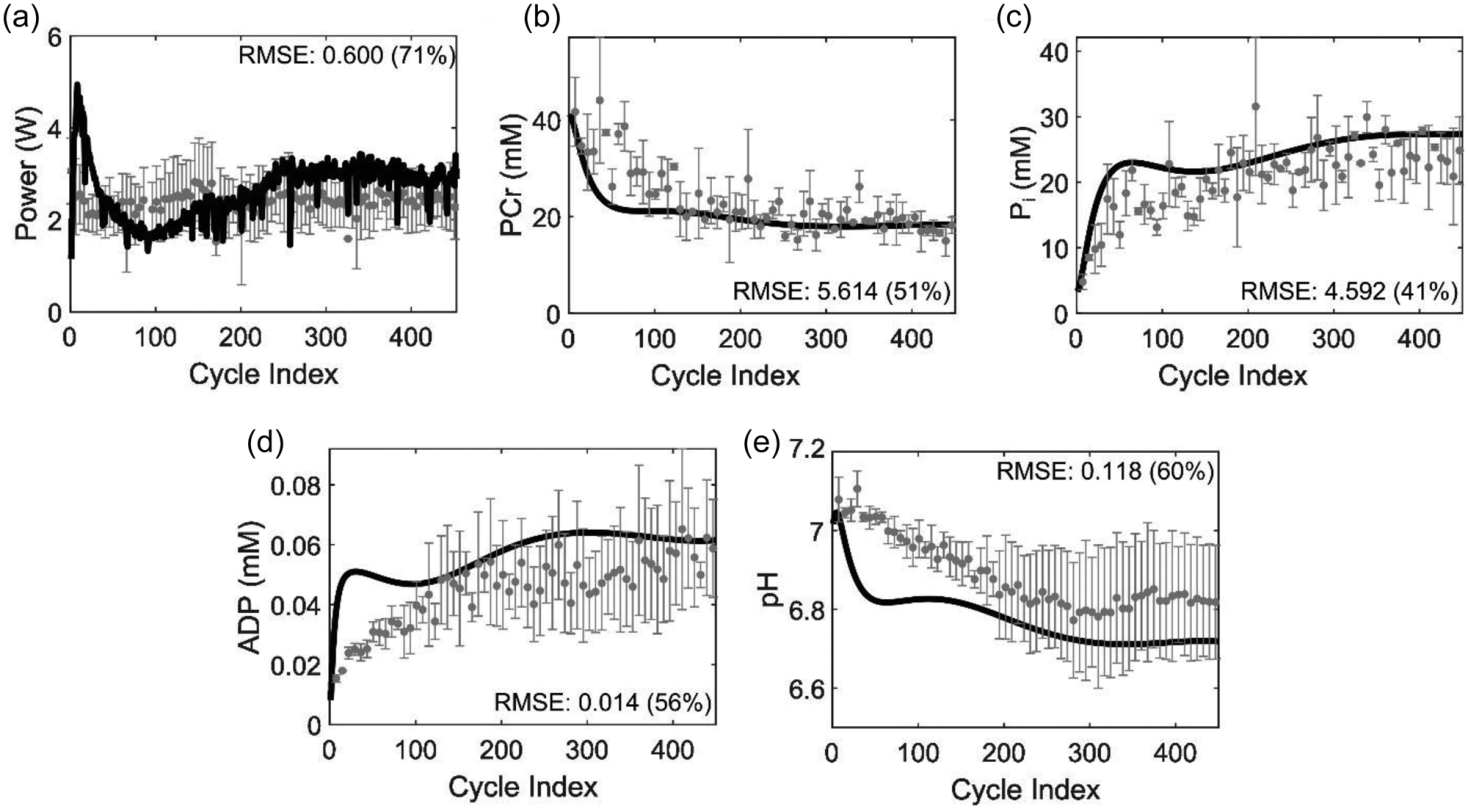
Cross‐bridge model predictions of dynamic plantar flexion exercise and validation with two additional subjects. (a) Model predictions of constant power generation using the integrated electromyography (EMG) data as input and comparison with experimentally recorded power data (circles with error bars). (b–e) Model predictions of the intramuscular metabolite alterations (continuous lines) compared with experimental data (circles with error bars) for phosphocreatine (PCr) (b), P_i_ (c), ADP (d), and H^+^ (e). Error bars indicate standard deviations (*n* = 2 subjects). RMSE, root mean square error.

To validate the proposed human skeletal muscle model, we further used the parameterized model to simulate the data collected from two independent subjects not included as part of the parameterization process. We used the average iEMG data (*n* = 2) and average sarcomere shortening velocity (*n* = 2) as inputs to simulate the dynamic plantar flexion cycles and compared the model simulations (continuous lines) with experimental data (circle with error bars) as shown in Figure 5. Table 3 shows the RMSE and fraction of predicted data points lying within 1 standard deviation of the experimental data. Our model predictions showed good agreement with the experimental data. For example, more than 50% of the model‐predicted points for power generation (Figure 5a), PCr (Figure 5b), ADP (Figure 5d) and pH (Figure 5e) were within 1 standard deviation of the experimental data points. For P_i_, we observed that 41% of the predicted data points were within 1 standard deviation of the experimental data points (Figure 5c). Overall, our model validation results showed that the RMSEs were within the experimental noise calculated between the two independent datasets, indicating that the model has the capability to predict alterations in muscle dynamics during a specific exercise protocol.

### 3.4 Effect of muscle activation potential and metabolite accumulation (ADP, H^+^ and P_i_) on force generation during plantar flexion exercise

**FIGURE 6:**
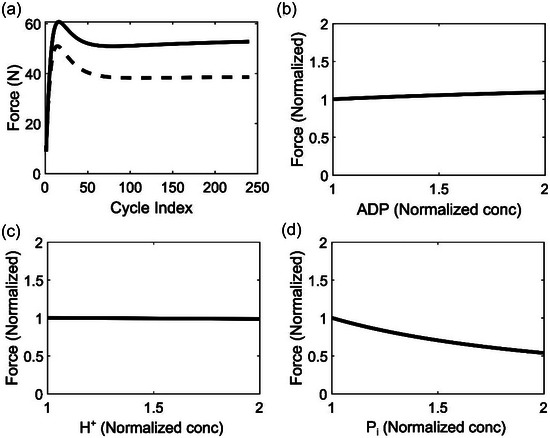
Model predictions for the effect of muscle activation potential and metabolite alterations (ADP, H^+^ and P_i_) on force generation. (a) Plot comparing force‐generating capacity simulated with measured iEMG profile (continous line) and force‐generating capacity simulated with a hypothetical constant basal activation (dotted line). (b–d) Plots showing the change in the normalized force with respect to relative change in ADP (b), H^+^ (c), and P_i_ (d) levels. We simulated plantar flexion cycles for 240 cycles and then continued for an additional 24 cycles at elevated concentrations of the metabolite under consideration while the rest of the metabolite levels were left unchanged. The *y*‐axis shows simulated force, at the end of 240 + 24 cycles, normalized by force required to move the piston during constant‐power plantar flexion exercise, and the *x*‐axis shows the concentration of metabolite normalized by the final concentrations recorded at the end of 240 cycles.

We used the validated model to study the effect of muscle activation potential, ADP, H^+^ and P_i_ on muscle force generation. Our experimentally measured EMG results showed an increasing muscle activation during the exercise (normalized iEMG data in Figure 3b). To understand the effect of this incremental muscle activation on force generation during the sustained plantar flexion exercise, we performed simulations with normalized iEMG remaining at the initial value (36%) throughout the exercise. These model simulations (dashed line in Figure 6a) showed that the subjects were not able to generate the required force early on and that force stayed consistently lower throughout the exercise as compared to that during the original experiment (continuous line, Figure 6a). These results suggest that the subjects would not be able to continue the exercise at the same intensity if they were not recruiting additional motor units or increasing their motor unit firing rate and may quit much earlier than the original experiment duration due to muscle fatigue.

To further understand the factors that might cause reduced force during exercise, we performed simulations to predict the change in force‐generating capacity with altered ADP, P_i_ or pH concentrations (Figure 6). We used the association/dissociation constants of these metabolites with different actin–myosin complexes to modulate the intramuscular metabolite levels and investigated whether their overaccumulation affected force generation. In the case of ADP, our model predicted only a marginal rise in force generation even after a two‐fold increase (Figure 6b) compared to the physiological concentration observed at the end of the plantar flexion exercise (Figure 3e). In comparison, we did not see any notable effect on generated force with a two‐fold increase in H^+^ concentration (Figure 6c). However, we observed a notable decrease in force generation with an overaccumulation of P_i_ (Figure 6d). Our simulations showed that the normalized force dropped to 30% when P_i_ increased by 50% and dropped to 47% when P_i_ accumulated 100% above its physiological concentration at the end of the plantar flexion exercise. The results show that the intramuscular accumulation of P_i_ is one of the major factors that inhibits the force‐generating capacity during exercise.

### 3.5 Skeletal muscle force‐generating capacity is sensitive to the P_i_ dissociation step during cross‐bridge cycling

**FIGURE 7:**
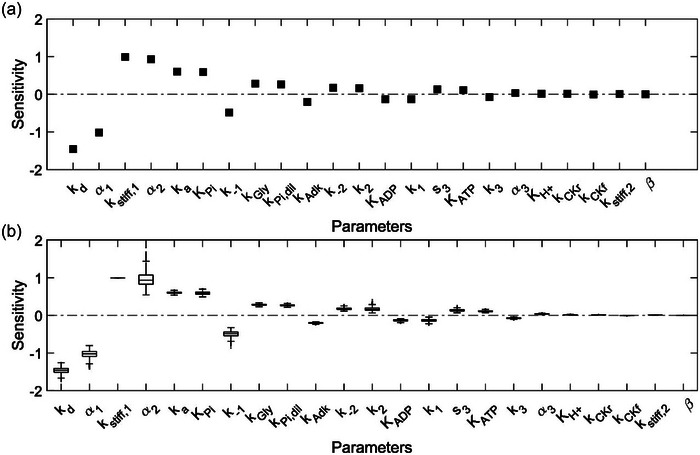
Parameter sensitivity analysis. Plots of local (a) and global (b) sensitivities calculated for all 23 parameters in the model. For the global analysis, we sampled 10,000 random parameter sets around the estimated optimal parameters by allowing for 10% variation in individual parameter values. We calculated the force sensitivities for each parameter for all 10,000 parameter sets and summarized them as box plots.

To understand the mechanism through which P_i_ inhibits force generation, we performed a sensitivity analysis to identify the parameters that impact a specific model output. This technique also evaluated the robustness of the model with regard to parameter estimation uncertainties. Specifically, we were interested in the parameters that most impact muscular force generation. Therefore, we performed both local (Figure 7a) and global (Figure 7b) sensitivity analyses with respect to force generation for the 23 model parameters we estimated using experimental data. Both the local and global sensitivity values were within the −2 to 2 range. Furthermore, of the 15 parameters found to impact cross‐bridge kinetics, both analyses revealed *k*_d_, α_1_, α_2_, *k*_a_ and *K*_Pi_ as the top five parameters to which force was most sensitive. Interestingly, three (*k*_d_, *k*_a_ and *K*_Pi_) of the five parameters were associated with the cross‐bridge cycle P state to A_1_ state transition. Of the parameters controlling the interactions between the cross‐bridge cycle and metabolite levels, force generation was at least four times more sensitive to *K*_Pi_ than others (*K*_ATP_, *K*_ADP_ and *K*_H_ *_+_*), which agrees with our earlier observation that P_i_ inhibits force generation significantly compared to other metabolites accumulated during plantar flexion exercise. Of the five metabolic parameters in the model, we found that force was sensitive only to *k*_Gly_, *k*_Pi,dil_ and *k*_adk_, and these observations were consistent between the local and global sensitivity analyses.

## 4 DISCUSSION

To understand how physiological factors, such as muscle activation and metabolite accumulation, affect force generation, we developed a computational model that combines a five‐state actin–myosin cross‐bridge cycle with key metabolic processes associated with skeletal muscle energetics. We used a mathematical implementation of Huxley's sliding filament theory (Huxley, 1957), used in the study by Tewari et al. (2016), to account for the cross‐bridge cycle. This implementation consisted of five cross‐bridge states and accounted for the effect of ADP, ATP and P_i_ on the kinetics of the cross‐bridge cycle. We updated the model by integrating metabolic processes, such as creatine kinase, glycolysis, adenylate kinase and intracellular phosphate buffering of pH, using mass action kinetics. We parameterized and validated the model using experimentally measured muscle activation signals and intramuscular metabolite alterations (PCr, P_i_, ADP and H^+^) collected as human participants performed a dynamic plantar flexion exercise at ∼20% above their critical power.

Our strategy of combining a five‐state cross‐bridge model with a kinetic model representing metabolic processes that are specific for skeletal muscles enabled us to leverage both force and metabolomics data from exercising humans for model parameterization and validation. The resultant skeletal muscle cross‐bridge model not only could simulate force generation but also recapitulated the dynamics of ADP, P_i_, PCr and pH during a plantar flexion type of exercise. Although the skeletal muscle cross‐bridge (Herzog & Schappacher‐Tilp, 2023) and metabolic models (Lai et al., 2008; Lambeth & Kushmerick, 2002; Lopez et al., 2020) were developed previously, they have rarely been used together in the context of muscle fatigue. This study demonstrates that by combining these two frameworks we can take advantage of non‐invasively collected time course metabolomics data from exercising humans to understand muscle fatigue development. Furthermore, the model also allowed us to evaluate the effect of different physiological factors, such as muscle activation and metabolic concentration, on skeletal muscle force‐generating capacity.

Surface EMG provides an indirect measure of muscle activation in exercising skeletal muscles. In our study, we observed that normalized integrated surface EMG increased over the exercise period, indicating a progressive recruitment of muscle motor units or increased motor unit firing rate by the neuromuscular system. Using a mathematical model, Contessa et al. (2016) reported a similar observation and hypothesized that the increased motor unit firing rate compensated for the decreased force generation from fatigued muscles. Our studies simulating constant muscle activation also supported this hypothesis (Figure 6a) since the model predicted a reduced force generation early into the exercise. Thus, in agreement with earlier studies, this study attests to the important role the neuromuscular system plays in providing a compensatory stimulation to mitigate the effects of early peripheral muscle fatigue development.

In our simulations investigating the effects of the accumulation of various metabolic species, we found that P_i_ accumulation greatly impacted the force generated by the skeletal muscles, which agrees with earlier studies where elevated P_i_ concentrations (25–30 mM) reduced peak isometric force by 5–19% in Ca^2+^‐activated rabbit and rat fibres (Coupland et al., 2001; Debold et al., 2004). The five‐state cross‐bridge model used in this study considers the release of P_i_ from the weakly bound state (A_1_) as a prerequisite for reaching the strongly bound state (A_2_) (see Figure 2 for details) (Kawai et al., 1993; Muangkram et al., 2020; Pate & Cooke, 1989; Tewari et al., 2016). A consequence of this assumption is that both the rate constants of the A_1_→P transition ($k_{d}$) and the A_1_→A_2_ transition ($k_{1}$) are modulated by P_i_ concentration and *K*_Pi_. Our model uses mass action‐ and rapid equilibrium‐based formulations of substrate inhibition kinetics to capture this relationship (Equations 12 and 13). Our sensitivity analysis indicated that force was sensitive to both $k_{d}$ and $K_{\mathrm{Pi}}$. In fact, they were among the top five parameters to which force was most sensitive, with $k_{d}$ being number 1. This in combination with the lack of sensitivity for $k_{1}$ indicates that, in our model, P_i_ impacts force by increasing the rate of cross‐bridge detachment by promoting the A_1_→P transition. Other models of cross‐bridge cycling assume that P_i_ release occurs after the actomyosin cross‐bridge transitions from the weakly bound state to the strongly bound state (Debold, 2021; Hibberd et al., 1985; Linari et al., 2010; Takagi et al., 2004). Simulations based on these models can also reproduce the P_i_‐mediated inhibition of force in skeletal muscle fibres (Dantzig et al., 1992) and in isolated myosin molecules (Debold et al., 2013). There are other indirect mechanisms through which P_i_ might inhibit the cross‐bridge kinetics. One proposed mechanism is that P_i_ directly interacts with the ryanodine receptor (RyR) on the surface of the sarcoplasmic reticulum (SR) and modulates Ca^2+^ release (Duke & Steele, 2001). Another potential mechanism is that Ca^2+^–P_i_ precipitation directly reduces the Ca^2+^ content in the SR (Westerblad & Allen, 1996). The model currently does not account for these mechanisms due to the lack of real‐time data on intracellular Ca^2+^ dynamics. Therefore, it is difficult to rule out the contribution of these mechanisms to fatigue development in this study.

Our simulations showed only a marginal effect on force for a two‐fold higher ADP accumulation than what was experimentally observed (Figure 6b). This indicates that ADP accumulation may not play a significant role in the development of muscle fatigue. Indeed, studies with transgenic mice accumulating up to ∼1.5 mM ADP in their skeletal muscles have shown that ADP does not affect normal force production (Hancock et al., 2005). For [H^+^], the model showed no effect even when the concentration was doubled (pH 6.43). This contradicts some of the experimental studies that observed a 10–20% decline in force even for pH ranges of 6.5–6.6 (Westerblad et al., 1997; Woodward & Debold, 2018). While the exact mechanism remains unknown, it has been hypothesized that H^+^ slows down the release of ADP from the A_3_ state (Figure 2) (Debold et al., 2008, 2011; Jarvis et al., 2018). Although we incorporated this hypothetical mechanism into our model, the simulations still predicted no effect on force production under elevated [H^+^].

**FIGURE 8:**
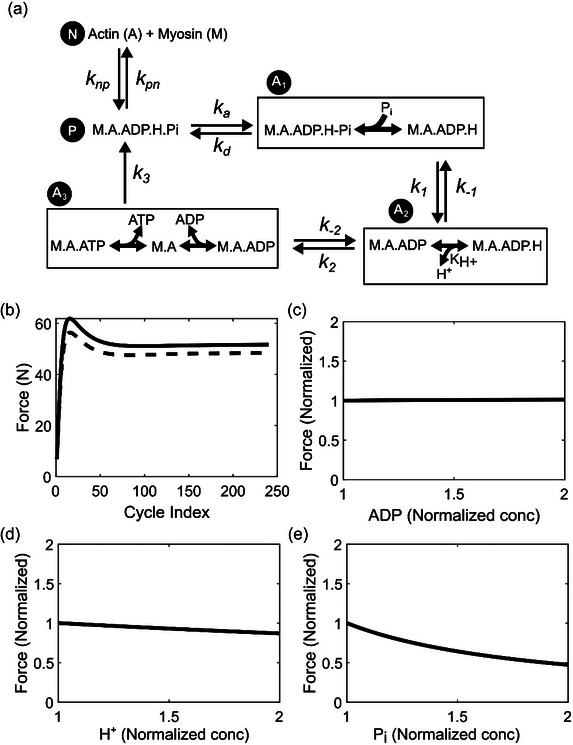
Evaluation of an alternate cross‐bridge mechanism where H^+^ was released from the A_2_ state as opposed to from the A_3_ state. (a) Schematic depiction of the modified cross‐bridge model that uses an alternative mechanism to explain proton (H^+^)‐mediated inhibition of cross‐bridge cycling and force generation. In this mechanism, H^+^ dissociation is a prerequisite for the A_2_→A_3_ transition, and therefore the rate of this transition is a function of the H^+^ dissociation constant (*K*_H+_). (b) Plot comparing force‐generating capacity simulated with a measured iEMG profile (continuous line) and with a hypothetical constant basal activation (dotted line). (c–e) Plots showing the change in the normalized force with respect to change in ADP (c), H^+^ (d), and P_i_ levels (e).

In order to test other potential mechanisms that simulate H^+^ inhibition of force production, we explored an alternative cross‐bridge‐based mechanism (Figure 8a) where the H^+^ from ATP hydrolysis remained attached to myosin until it was released in the A_2_ state and this H^+^ dissociation was a prerequisite for the A_2_→A_3_ transition. Parameterizing this model provided comparably good fits (Figure S2), although there was a slight increase in RMSE values compared to the original model. Interestingly, the modified model indicated force inhibition with increased H^+^ concentration (Figure 8d), and also showed a similar behaviour for the perturbations in EMG (Figure 8b), ADP (Figure 8c) and P_i_ (Figure 8e) compared to the original model. This indicates the usefulness of our computational model to generate hypothetical mechanisms that can explain the observed metabolite‐mediated inhibition of force generation in muscles. Overall, these results show some gaps in our understanding of the interaction between H^+^ and cross‐bridge cycles as well as the complexity involved in developing skeletal muscle models and the limitations associated with them. For example, one of the proposed mechanisms through which H^+^ affects muscle force generation is by modulating the Ca^2+^/troponin‐mediated activation of muscle fibres (Nelson & Fitts, 2014; Parsons et al., 1997; Unger & Debold, 2019). Other possible mechanisms include H^+^‐mediated modulation of ATPase activity associated with myofibrils and Ca^2+^ transporters on the SR membrane and sarcolemma. However, due to challenges in accurately measuring intracellular Ca^2+^ dynamics during exercise, we were not able to incorporate them in our current model formulation.

We also note that the model in its current form does not segregate the metabolite pools into different organelles/compartments, such as the mitochondria and cytoplasm, as found in musculoskeletal cells. Such compartmentalization, as used in recent modelling studies (Lopez et al., 2020), would allow us to study the effect of energy shuttle reactions, such as creatine kinase, on force generation. Furthermore, due to challenges in obtaining Ca^2+^ concentration measurements in exercising humans, the current model uses EMG data as an overall indication of muscle stimulation. Modelling Ca^2+^‐mediated activation of skeletal muscles would facilitate the evaluation of other potential mechanisms through which H^+^ and P_i_ are hypothesized to modulate calcium dynamics and thereby impact force generation. Such potential mechanisms include but are not limited to: (1) modulation of Ca^2+^‐troponin interaction by H^+^ (Nelson & Fitts, 2014), (2) modulation of myofibrillar ATPases by H^+^ (Fitts, 2016), (3) H^+^‐mediated modulation of ATPase activity of Ca^2+^ channels on the SR membrane (Wolosker et al., 1997) and Na^+^–K^+^ channels on the sarcolemma (Fitts, 2016), (4) P_i_‐induced inhibition of RyR (Duke & Steele, 2001), and (5) Ca^2+^‐P_i_ precipitation in the SR (Westerblad & Allen, 1996).

### 4.1 Conclusion

We developed a computational skeletal muscle model that accounts for cross‐bridge cycling and key metabolic processes of contractile function. We parameterized and validated the model using force, surface EMG, and metabolite concentration data collected non‐invasively from human participants performing a plantar flexion exercise above their critical power to task failure. The model predicted that the observed increase in muscle activation during exercise compensates for the fatigue‐induced force reduction and that P_i_ accumulation affects force production by increasing the rate of detachment of actin and myosin at the weakly bound state. Furthermore, the model predicted that accumulation of ADP only had a minimal effect on force in agreement with literature reports. Using the proposed cross‐bridge cycling model, we explored alternative mechanisms to explain the observed inhibitory effect of H^+^ accumulation on force generation. However, we need further studies exploring the role of H^+^ in cross‐bridge kinetics to better understand how H^+^ contributes to muscle force generation and fatigue development.

## AUTHOR CONTRIBUTIONS

Conceptualization: John I. Hendry, Gwenael Layec, Edward P. Debold, Shivendra G. Tewari, Anders Wallqvist, and Venkat R. Pannala; Experimental data collection, curation, processing, and quality control: Muhammet Enes Erol and Gwenael Layec; Modelling, simulation, and analysis: John I. Hendry; Writing—original draft preparation: John I. Hendry; Writing—review and editing: John I. Hendry, Muhammet Enes Erol, Gwenael Layec, Edward P. Debold, Shivendra G. Tewari, Anders Wallqvist and Venkat R. Pannala; Supervision: Anders Wallqvist and Venkat R. Pannala; Funding acquisition: Anders Wallqvist. All authors have read and approved the final version of this manuscript and agree to be accountable for all aspects of the work in ensuring that questions related to the accuracy or integrity of any part of the work are appropriately investigated and resolved. All persons designated as authors qualify for authorship, and all those who qualify for authorship are listed.

## CONFLICT OF INTEREST

The authors declare that the research was conducted in the absence of any commercial or financial relationships that could be construed as a potential conflict of interest.

## Supporting information



Figure S1. Slack plot of the ^31^P spectra acquired from the gastrocnemius muscle of one of the participants while they performed the plantar flexion exercise.
Figure S2. Simulation of power generation and metabolite dynamics using the alternate model, where H^+^ release occurs in the A_2_ state.

## Data Availability

The models used in this study are publicly available at GitHub (https://github.com/BHSAI/Musculo_skeletal_model_dyanmic_plantar_flexion_exercise). All data will be made available following a written request to the corresponding author, along with a summary of the planned research.
