# Supplementary material for: A human skeletal muscle cross‐bridge model to characterize the role of metabolite accumulation in muscle fatigue
Source: Exp Physiol. 2025 May 31;110(9):1283–301. doi: 10.1113/EP092843 (PMC12400838; doi:10.1113/EP092843)
Supplement: Supplementary file 1 — Figure S1. Slack plot of the 31P spectra acquired from the gastrocnemius muscle of one of the participants while they performed the plantar flexion exercise. Figure S2. Simulation of power generation and metabolite dynamics using the alternate model, where H+ release occurs in the A2 state. [file EPH-110-1283-s001.docx]

**Supporting Information**

**A human skeletal muscle cross-bridge model to characterize the role of metabolite accumulation in muscle fatigue**

John I. Hendry^1,2^, Muhammet Enes Erol^3,4^, Gwenael Layec^3,4^, Edward P. Debold^3^, Shivendra G. Tewari^1,2^, Anders Wallqvist^1,*^, and Venkat R. Pannala^1,2,*^

^1^Department of Defense Biotechnology High Performance Computing Software Applications Institute, Defense Health Agency Research & Development, Medical Research and Development Command, Fort Detrick, MD 21702, USA

^2^The Henry M. Jackson Foundation for the Advancement of Military Medicine, Inc., Bethesda, MD 20817, USA

^3^Department of Kinesiology, University of Massachusetts, Amherst, MA 01003, USA

^4^School of Health and Kinesiology, University of Nebraska, Omaha, NE 68182, USA


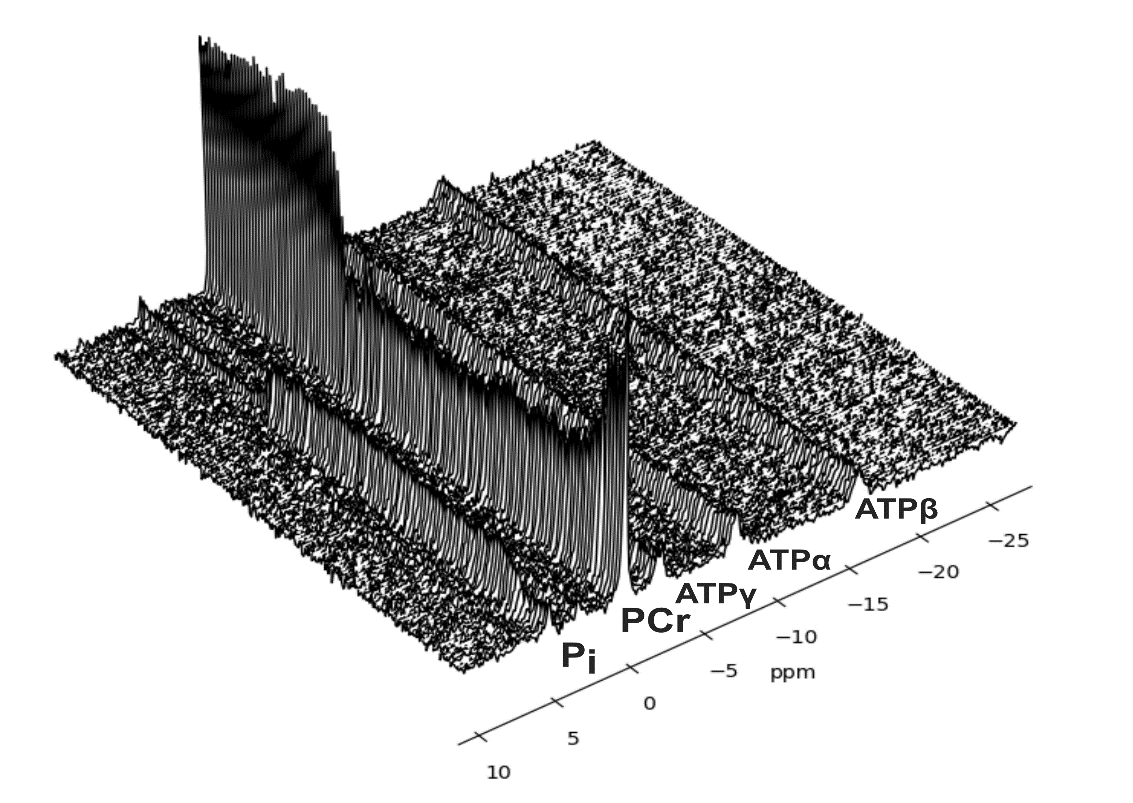


**Figure S1.** Slack plot of the ^31^P spectra acquired from the gastrocnemius muscle of one of the participants while they performed the plantar flexion exercise. P_i_: inorganic phosphate; PCr: phosphocreatine; ATP α, β, γ: phosphate groups of an ATP molecule.


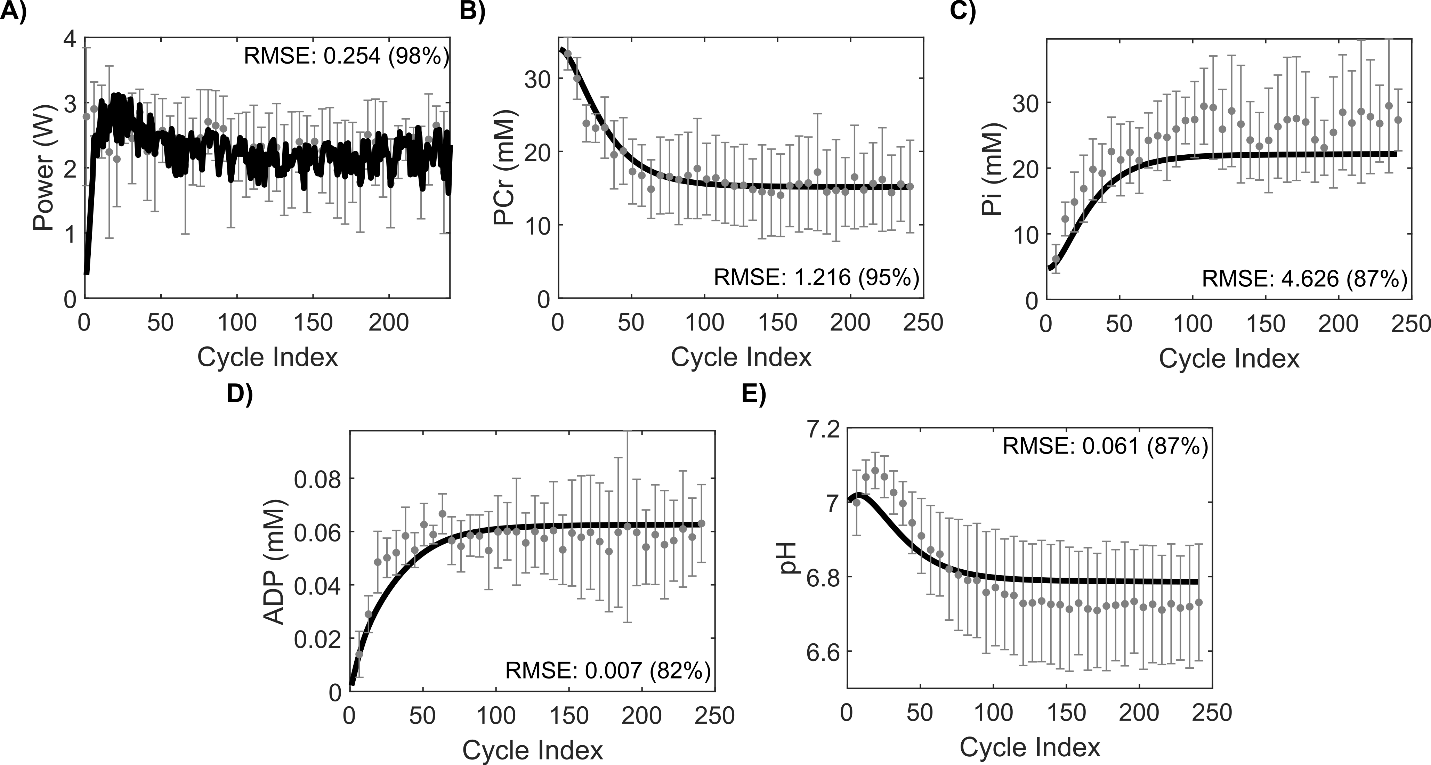


**Figure S2.** Simulation of power generation and metabolite dynamics using the alternate model, where H^+^ release occurs in the A_2_ state. A) Plots comparing experimental (solid circles) and simulated (continuous line) power for the modified cross-bridge model. B-E) Plots comparing experimental (solid circles) and simulated (continuous lines) levels of phosphocreatine (PCr) (B), P_i_ (C), ADP (D), and H^+^ (E) for the modified cross-bridge model. Error bars indicate standard deviations (N=5). P_i_: inorganic phosphate; PCr: phosphocreatine; RMSE: root mean square error.
